# Baicalein Inhibits the Invasion and Metastatic Capabilities of Hepatocellular Carcinoma Cells via Down-Regulation of the ERK Pathway

**DOI:** 10.1371/journal.pone.0072927

**Published:** 2013-09-06

**Authors:** Kunlun Chen, Shu Zhang, Yuanyuan Ji, Jun Li, Peng An, Hongtao Ren, Rongrui Liang, Jun Yang, Zongfang Li

**Affiliations:** 1 General Surgeon Department of Cadre’s Ward, the Second Affiliated Hospital, School of Medicine, Xi’an Jiaotong University, Xi’an, P. R. China; 2 National-Local Joint Engineering Research Center of Biodiagnostics & Biotherapy, Xi’an Jiaotong University, Xi’an, P. R. China; 3 Key Laboratory of Environment and Genes Related to Diseases of the Education Ministry, School of Medicine, Xi’an Jiaotong University, Xi’an, P. R. China; Institut für Pathologie, Germany

## Abstract

Baicalein, a widely used Chinese herbal medicine, has historically been used in anti-inflammatory and anti-cancer therapies. However, the anti-metastatic effect and molecular mechanism(s) of baicalein on hepatocellular carcinoma (HCC) remain poorly understood. Therefore, the purpose of this study was to assess the anti-metastatic effects of baicalein and related mechanism(s) on HCC. Based on assays utilized in both HCC cell lines and in an animal model, we found that baicalein inhibited tumor cell metastasis *in vivo* and *in vitro*. Furthermore, after treatment with baicalein for 24 hours, there was a decrease in the levels of matrix metalloproteinase-2 (MMP-2), MMP-9 and urokinase-type plasminogen activator (u-PA) expression as well as proteinase activity in hepatocellular carcinoma MHCC97H cells. Meanwhile, the expression of tissue inhibitor of metalloproteinase-1 (TIMP-1) and TIMP-2 were increased in a dose-dependent fashion. Moreover, baicalein treatment dramatically decreased the levels of the phosphorylated forms of MEK1 and ERK1/2. MEK1 overexpression partially blocked the anti-metastatic effects of baicalein. Combined treatment with an ERK inhibitor (U0126) and baicalein resulted in a synergistic reduction in MMP-2, MMP-9 and u-PA expression and an increase in TIMP-1 and TIMP-2 expression; the invasive capabilities of MHCC97H cells were also inhibited. In conclusion, baicalein inhibits tumor cell invasion and metastasis by reducing cell motility and migration via the suppression of the ERK pathway, suggesting that baicalein is a potential therapeutic agent for HCC.

## Introduction

As a common malignant neoplasm and a cause of cancer-related death in Asia and Africa [1], HCC is associated with a high rate of mortality [2] due to a lack of adequate treatment options to treat HCC invasion and metastasis. Based on observations made in the past few years, the prognosis of HCC is poor even in patients who have received what were thought to be curative treatments [3]. A lack of effective treatments against HCC invasion and metastasis has become the major obstacle to survival and quality of life in HCC patients [4].

Metastasis involves multiple processes and various cytophysiological changes, such as an alteration of the adhesive capability between cells and the degradation of the extracellular matrix (ECM). Several proteases, such as matrix metalloproteinases (MMPs), cathepsins and plasminogen activator (PA), enable cancer cells to degrade the ECM. MMP-2, MMP-9 and u-PA play important roles in degrading basement membranes and are intricately involved in cancer invasion and metastasis [5,6]. Nevertheless, tissue inhibitors of metalloproteinase (TIMPs) act through the formation of a tight and noncovalent complex with their cognate enzymes and are able to affect the biological activities of MMPs [7,8].

Increased activity of extracellular signal-regulated kinase1/2 (ERK1/2) has been observed in almost half of known human tumor cell lines and in a large number of human primary tumors derived from various origins [9]. It has been reported that invasion and metastasis by HCC cells requires specific intracellular signaling cascade activations among which the ERK signaling pathway is considered crucial [10–13].

**Figure 1:**
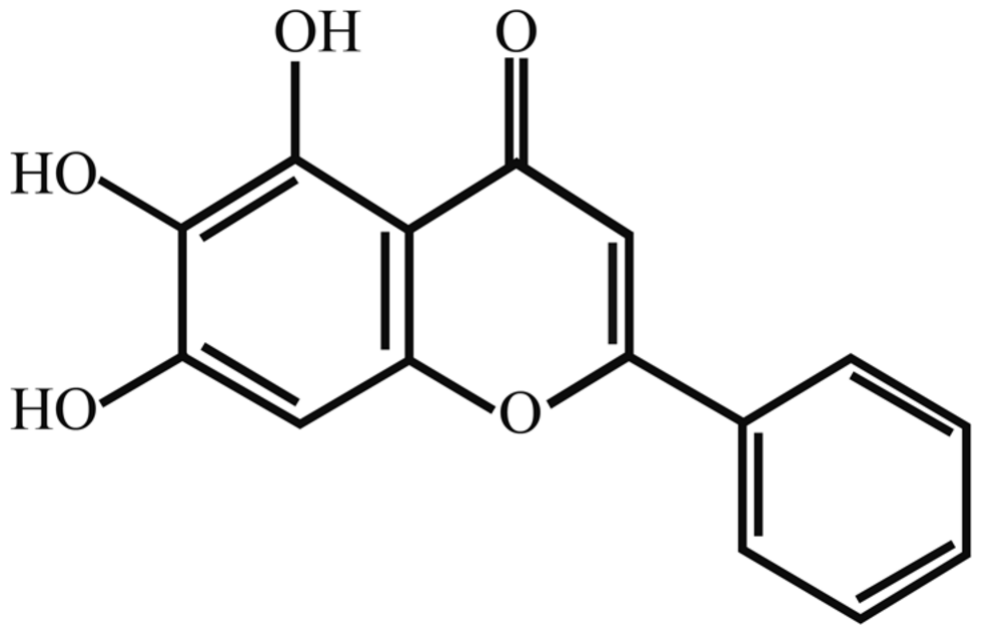
Chemical structure of baicalein.

Many plant-derived agents have been indicated to effectively inhibit invasion of human HCC cells [14]. Baicalein (5,6,7-trihydroxy-2-phenyl-4H-1-benzopyran‑4‑one), extracted from the roots of *Scutellaria* *radix* or *Scutellaria* *baicalensis* , is a purified flavonoid with a defined chemical structure (Figure 1). In our previous studies, baicalein exerted a significantly inhibitory effect on HCC cells (HepG2), while it had relatively little inhibitory effect on a normal liver cell line (HL-7702) [15]. Baicalein’s anti-tumor biological effect has been confirmed in various cancers, but the anti-metastatic effect and related mechanism(s) in HCC are still unknown. Thus, the present study was designed to investigate the effects and mechanisms of baicalein against HCC invasion and metastasis.

## Materials and Methods

### Ethics statement

Male BALB/c nude mice (4-week-old) were supplied by the Experimental Animal Center of Xi’an Jiaotong University, China. This study was carried out in accordance with the recommended guidelines for the care and use of laboratory animals issued by the Chinese Council on Animal Research. The protocol was approved by the ethics committee of Xi’an Jiaotong University.

### Reagents

Fetal bovine serum (FBS), penicillin and streptomycin were ordered from Gibco. Baicalein and U0126 were ordered from Sigma. Dulbecco’s modified Eagle’s medium (DMEM) was purchased from Invitrogen. Anti-Phospho-MEK1 (Thr386) (p-MEK1) and anti-MEK1 were purchased from Millipore Co. Anti-Phospho (Thr202/Tyr204) ERK1/2 (p-ERK1/2), anti-ERK1/2, anti-MMP-2, anti-MMP-9, anti-TIMP-1 and anti-TIMP-2 antibodies were purchased from Cell Signaling. Anti-u-PA antibody and anti-β-actin was purchased from Santa Cruz.

### Cell culture

The human HCC cell line MHCC97H was purchased from the Liver Cancer Institute of Fudan University (Shanghai, China). The cells were cultured in DMEM supplemented with 10% FBS, 100 U/ml penicillin, 100 μg/ml streptomycin and 2 mmol/l glutamine. All cells were incubated at 37^°^C with 5% CO_2_.

### Construction of expression plasmids and transfection

The construction of the expression plasmids and their transfection were performed as previously described [15]. Briefly, we made the full-length pcDNA3.1 (Invitrogen) MEK1 vector by cloning the full-length PCR product of MEK1 with KOD® DNA polymerase (Toyobo, Osaka, Japan). We used DNA sequencing to confirm the plasmid sequences. For transient transfection experiments, cells were plated 24 h before transfection in a 6-well plate at a density of 2×10^5^ cells per well. For the transfection, we used Lipofectamine 2000 (Invitrogen) with 4.0 μg pcDNA3.1(+)-MEK1 vector or 4.0 μg pcDNA3.1(+) empty vector (as a negative control) in accordance with the manufacturer’s protocol.

### Assessment of cell viability

Cell viability was determined by a colorimetric 3-(4,5-dimethylthiazol-2-yl) 2,5-diphenyltetrazolium bromide (MTT) assay in accordance with previously described protocols [16]. Briefly, we plated the cells in 96-well culture plates (2×10^4^ cells per well). The cells were treated for 24 h with serially diluted concentrations of baicalein. The cells were then washed twice with PBS and incubated with 5 mg/ml MTT (Sigma) for 4 h. The living cells absorbed the reagent and eventually produced an insoluble blue formazan product. After the incubation period, the cells were washed with PBS and then solubilized with dimethyl sulfoxide (DMSO). The optical density was read in an enzyme-linked immunosorbent assay plate reader.

### Wound healing assays

MHCC97H cells were seeded into a 6-well plate and cultured to 70% confluency in medium containing 10% FBS. Cell monolayers were wounded by a plastic tip (1 mm) that touched the plate as described [17]. MHCC97H cells were then incubated in serum-containing medium (2% serum) with baicalein (0, 10, 20 and 30 µM) for 24 h. Pictures were taken at 0, 6, 12, 18 and 24 h after baicalein was added. The migration distance of the cells was measured at three different sites using Photoshop software (Adobe). The percent migration rate was expressed as a percentage of the control.

### In vitro invasion assays

The *in vitro* invasion assay was performed using the Bio-Coat Matrigel invasion assay system (BD) as described previously [14]. MHCC97H cells were pretreated with 0, 10, 20 and 30 µM baicalein or U0126 (10 µM) for 24 h. Cells were suspended in serum-free DMEM medium and seeded into the upper chambers; FBS (10%) was added to the bottom chambers. After 24 h, the cells on the upper side were removed with a cotton swab, while the cells on the bottom side of the filter were fixed, stained and counted. For transfection experiments, the cells were seeded 24 h after transfection. The percent invasive rate was expressed as a percentage of control.

### Establishment of the orthotopic transplanted nude mouse model of HCC metastasis

We subcutaneously injected MHCC97H cells (1×10^7^ cells/animal) into nude mice to produce implanted tumors. The length and width of tumor were measured with a slide caliper (volume = (length ×width^2^)/2) [18]. When the tumor diameter grew to approximately 0.5 cm to 0.7 cm (20 days), the tumors were dissected into pieces of approximately 2×2×1 mm^3^. The tumors were re-inoculated into the liver parcel of different nude mice [19]. The control group (n=10) received diluent vehicle treatment only, whereas the treatment group (n=10) received baicalein (10 mg/kg/day) via oral administration. All of the animals that were inoculated intrahepatically were sacrificed for the metastatic assay. To examine the presence of metastases, the lung and liver were removed after inoculation for 35 days. The organs were fixed in 10% neutral formalin for paraffin-embedded sections [20]. We counted the number of metastases in the lungs after hematoxylin and eosin (HE) staining.

### Quantitative real-time RT-PCR

Total RNA was isolated using the RNeasy Mini kit (Invitrogen). cDNA was synthesized with SuperScript III Reverse Transcriptase (Invitrogen). Quantitative real-time RT-PCR (qRT-PCR) was performed using SYBR Green II in accordance with the PrimeScript RT-PCR Kit protocol (TaKaRa). The specific primers that were used are shown in Table 1. β-actin was used as internal control. The analysis of the relative gene copy number data for MMP-2, MMP-9 and u-PA was performed using the 2^-ΔΔCt^ method.

**Table 1:** Primers used for qRT-PCR analysis.

Gene	Primer sequence	Accession number	Expected size(bp)
MMP-2	5’-CTCATCGCAGATGCCTGGAA-3’	NM_001127891.1	104
	5’-TTCAGGTAATAGGCACCCTTGAAGA-3’		
MMP-9	5’-GTCCACCCTTGTGCTCTTCC-3’	NM_004994.2	94
	5’-GCCACCCGAGTGTAACCAT-3’		
u-PA	5’-ATCTGCCTGCCCTCGATGTATAA-3’	NM_001145031.1	108
	5’-TTTCAGCTGCTCCGGATAGAGATAG-3’		
β-actin	5’- CCATCGTCCACCGCAAAT-3’	NM_001101.3	104
	5’- CCATCGTCCACCGCAAAT-3’		

### Zymography

Cells were treated with different concentrations of baicalein or U0126 at 37°C for 24 h, and samples of conditioned media were collected. Appropriate volumes of the unboiled samples (adjusted by vital cell number) were separated by 0.1% gelatin-8% SDS-PAGE electrophoresis. After electrophoresis, the gels were washed twice in 2.5% Triton X-100 at room temperature for 30 min and then incubated in reaction buffer (10 mM CaCl2, 40 mM Tris-HCl and 0.01% NaN3, pH 8.0) at 37°C for 12 h. Coomassie brilliant blue R-250 gel stain was then used to stain the gel. The intensities of bands on the gels were calculated using an image analysis system (Bio-Rad Laboratories, Richmond, CA). For the determination of u-PA, 20 mg/ml plasminogen and 2% casein (w/v) were added to an 8% SDS-PAGE gel as described in the gelatin zymography.

### Western blotting analysis

After treatment with different concentrations of baicalein or U0126, 1×10^6^ cells were suspended in 250 μl of lysis buffer (40 mmol/l Tris-HCl, 1 mmol/l EDTA, 150 mmol/l KCl, 100 mmol/l NaVO3, 1% Triton X-100, 1 mmol/l PMSF, pH 7.5). The proteins (50 μg) were separated by 10% SDS-polyacrylamide gel electrophoresis and transferred onto PVDF membranes. The membranes were subsequently blocked in defatted milk (5% in Tris-buffered saline with TWEEN-20(TBST) buffer) at 37^°^C for 1 h to block non-specific binding and were then incubated overnight with antibodies against MEK1, p-MEK1, ERK1/2, p-ERK1/2, MMP-2, MMP-9, u-PA, TIMP-1, TIMP-2 or β-actin in TBST containing 5% defatted milk at 4^°^C. The membranes were then incubated with a horseradish peroxidase goat anti-mouse or anti-rabbit IgG antibody for 1 h at room temperature. The bands were detected with an enhanced chemiluminescence kit (Amersham, ECL Plus, Freiburg, Germany) and exposed by autoradiography. The densitometric analysis was performed using ImageJ software (GE Healthcare, Buckinghamshire, UK), and the results were expressed as arbitrary units (a.u.).

### Statistical analysis

Experiments were repeated three times, and dates were analyzed using Student’s *t*-test. All statistical tests and corresponding *p*-values were two sided. *p*<0.05 was considered to be statistically significant. We performed correlation analysis by the Z-test.

## Results

### Baicalein inhibits the proliferation of MHCC97H cells

**Figure 2:**
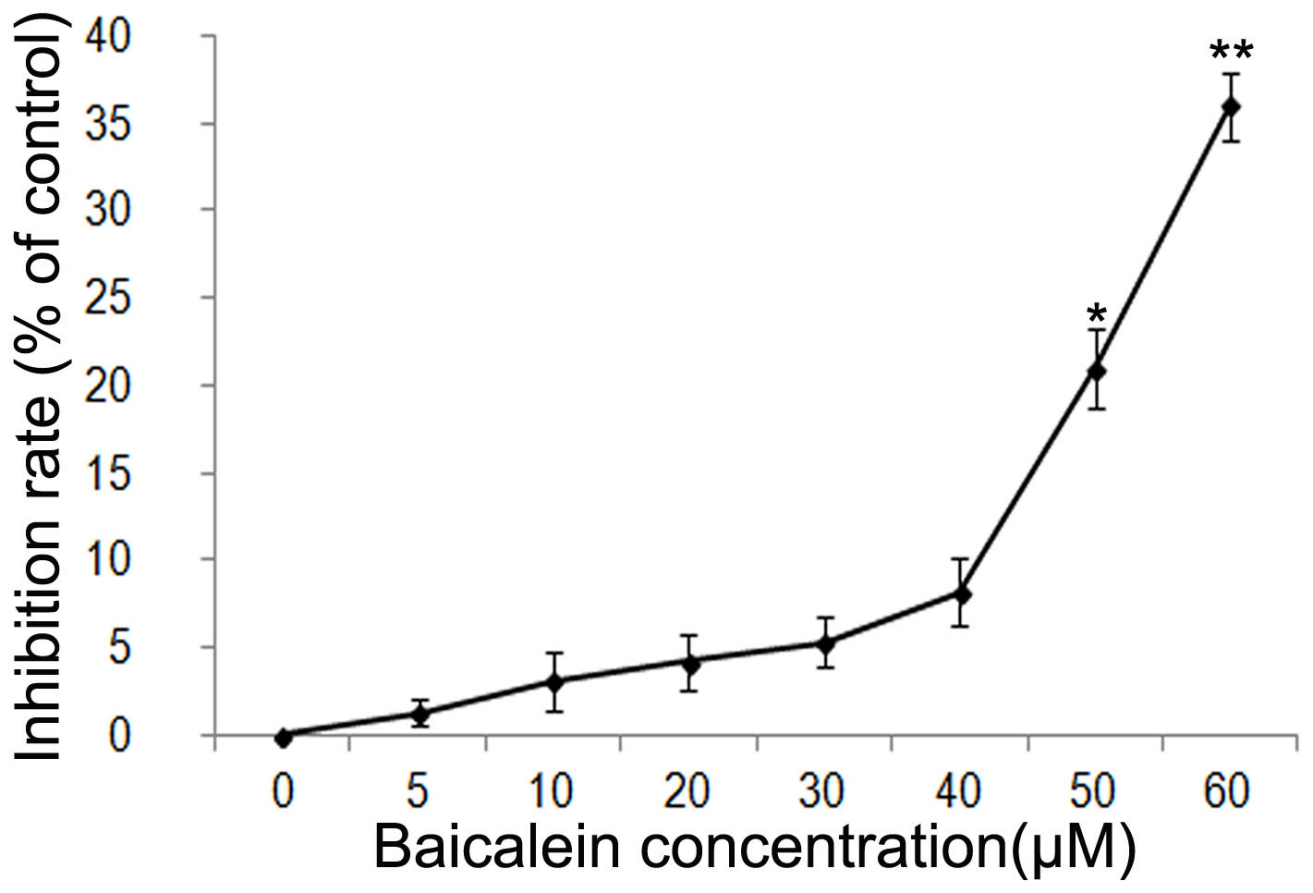
Baicalein inhibits the proliferation of MHCC97H cells. Cell viability was measured by MTT assay. Values represent the means ± standard deviation (SD) of three independent experiments performed in triplicate. *p < 0.05 and **p < 0.01 compared with the control group.

The anti-proliferation effects of baicalein at various concentrations (0 to 60 μM) on MHCC97H cells are shown in Figure 2. At 50 μM, baicalein significantly inhibited the proliferation of MHCC97H cells. At concentrations below 50 μM, the anti-proliferative effect was not obvious; thus, we chose a concentration range of baicalein lower than this for all subsequent experiments.

### Baicalein inhibits the motility and invasion of MHCC97H cells

**Figure 3:**
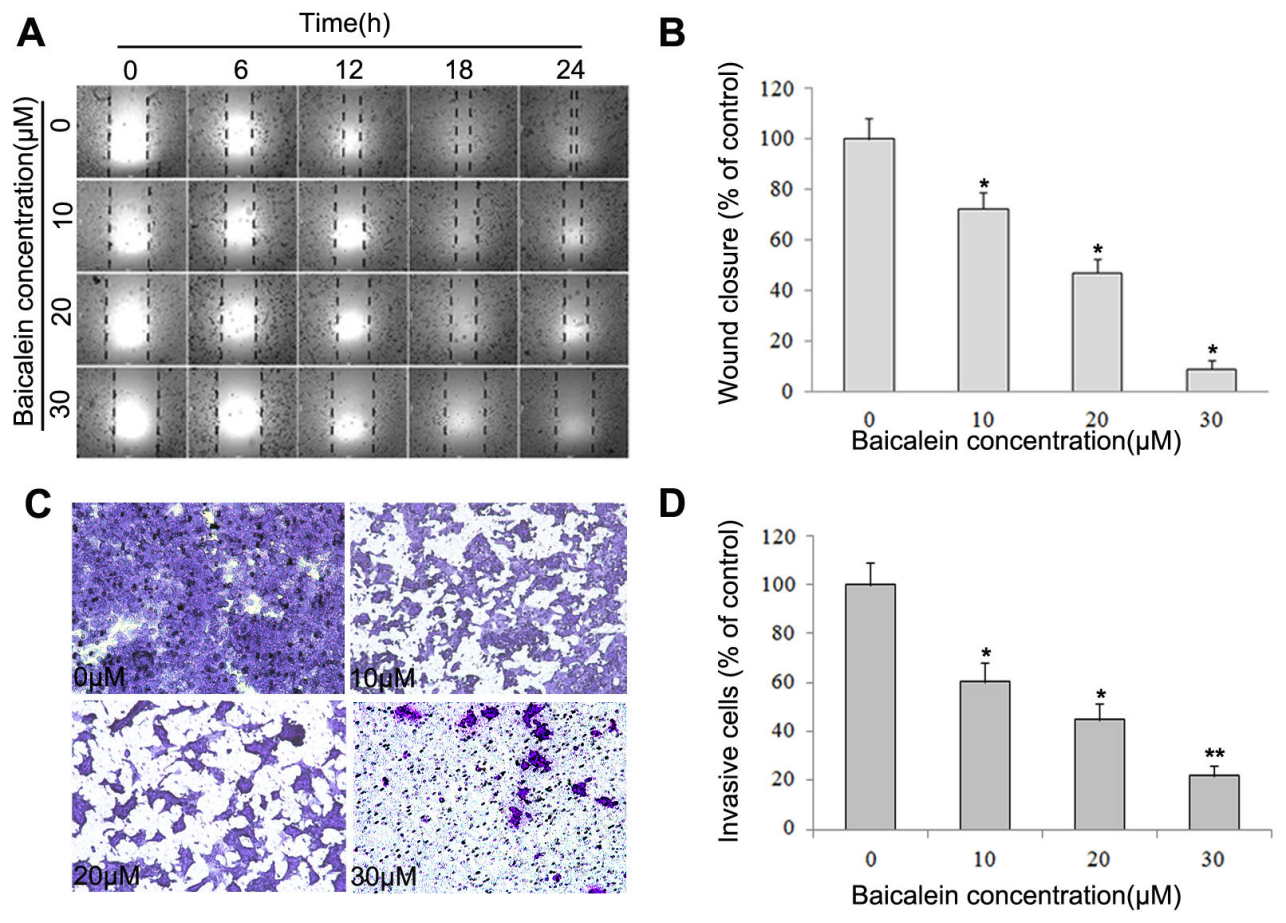
Baicalein inhibits the motility and invasiveness of MHCC97H cells. (A) MHCC97H cells were wounded and then incubated in media containing 2% serum with varying concentrations of baicalein (0, 10, 20 and 30 µM) for 24 h. Pictures were taken at 0, 6, 12, 18 and 24 h after addition of baicalein. (B) The percent migration rate is expressed as a percentage of the control (0 µM). (C) MHCC97H cells were pretreated with 0, 10, 20 and 30 µM baicalein for 24 h and were then seeded in the upper wells. FBS (10%) was added to the bottom chambers for 24 h to induce cell invasion. After 24 h, cells on the bottom side of the filter were fixed, stained and counted. (D) The percent invasion rate was expressed as a percentage of the control (0 µM). Values represent the means ± SD of three independent experiments performed in triplicate. *p < 0.05 and **p < 0.01 compared with the control group.

Because cell motility is a measure of the metastatic potential of cancer cells, the motility of MHCC97H cells was examined. As shown in Figure 3A, a continuous rapid movement was observed in control cells. The movement of MHCC97H cells was significantly reduced by treatment with baicalein in a concentration-dependent manner; the inhibition rate was approximately 27.5%, 52.9% and 91.1% at 24 h with 10 µM, 20 µM and 30 µM baicalein, respectively (Figure 3 B). Figure 3C shows the effect of baicalein on the invasiveness of MHCC97H cells that were treated with 0, 10, 20 and 30 µM of baicalein for 24 h. Baicalein reduced the invasion of MHCC97H cells substantially in a concentration-dependent manner. Similar anti-metastatic effect of baicalein was observed in HepG2 cells (dates not shown). Quantification analysis indicated that the invasiveness of MHCC97H cells was reduced by 39.7%, 55.1% and 78.1% when cells were treated with 10 µM, 20 µM and 30 µM of baicalein (Figure 3D), respectively.

### Inhibition effect of baicalein on the transcriptional levels of MMP-2, MMP-9 and u-PA

**Figure 4:**
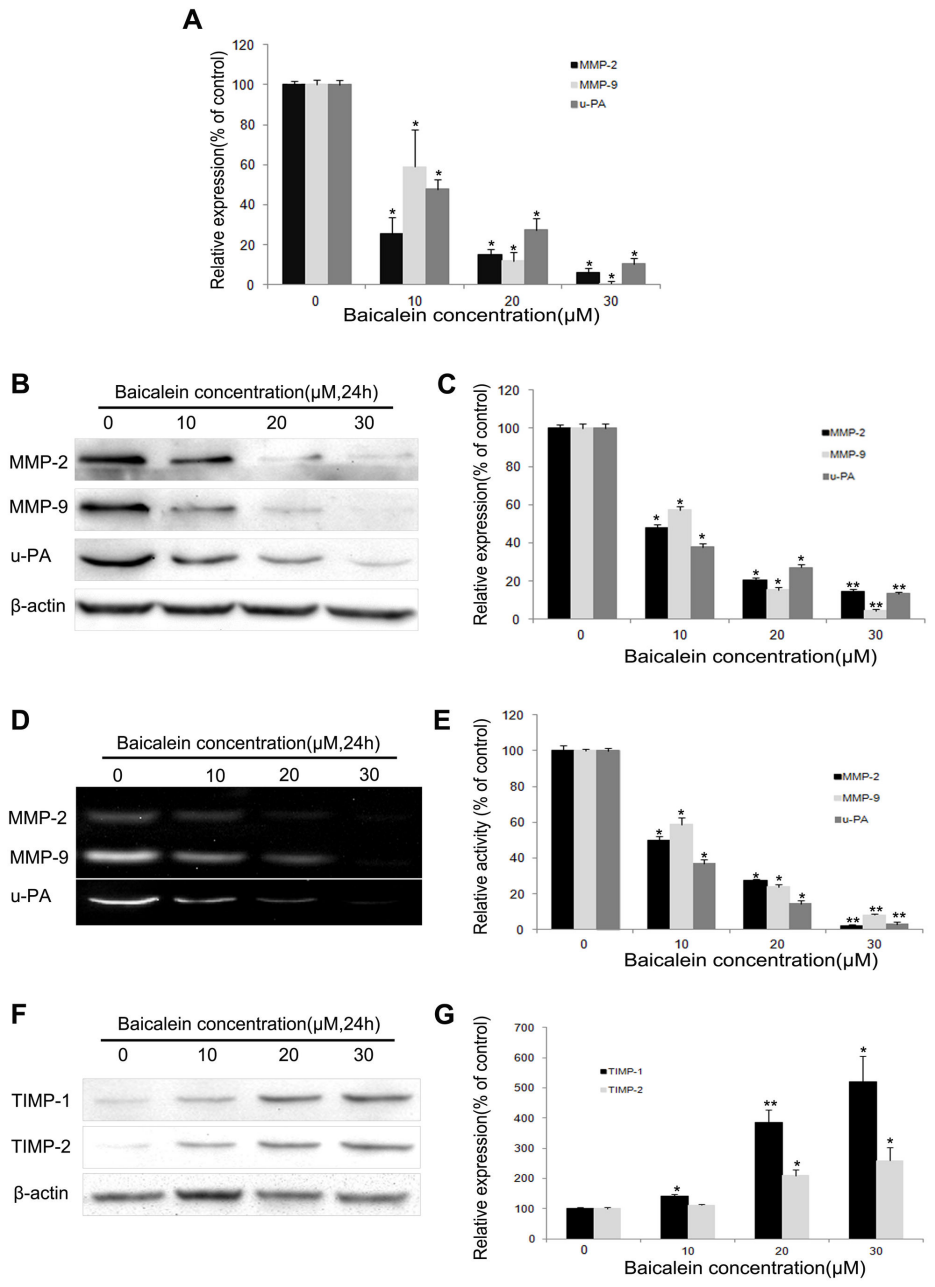
Baicalein suppresses the expression and activity of MMP-2, MMP-9 and u-PA and promotes the expression of TIMP-1 and TIMP-2 in MHCC97H cells. (A) The effects of baicalein on the expression levels of MMP-2, MMP-9 and u-PA mRNA were assessed by qRT-PCR. (B) MHCC97H cells were treated with baicalein (0, 10, 20 and 30 µM) for 24 h and then subjected to western blotting to analyze the protein levels of MMP-2, MMP-9 and u-PA. (C) Quantification of the protein levels of MMP-2, MMP-9 and u-PA. (D) Effects of baicalein on the activities of MMP-2, MMP-9 and u-PA. (E) Quantification of the activities of MMP-2, MMP-9 and u-PA. (F) MHCC97H cells were treated with baicalein (0, 10, 20 and 30 µM) for 24 h and then subjected to western blotting to analyze the protein levels of TIMP-1 and TIMP-2. (G) Quantification of the protein levels of TIMP-1 and TIMP-2. Values represent the means ± SD of three independent experiments performed in triplicate. *p < 0.05 and **p < 0.01 compared with the control group.

We used qRT-PCR to investigate the inhibitory effect of baicalein on MMP-2, MMP-9 and u-PA in MHCC97H cells. MHCC97H cells were treated with 0, 10, 20 and 30 µM baicalein for 24 h and then subjected to qRT-PCR. We found that baicalein could significantly reduce the transcriptional levels of MMP-2, MMP-9 and u-PA in a concentration-dependent manner (Figure 4A). The inhibition rate of MMP-2 was approximately 74.81%, 85.19% and 94.49% after 24 h of treatment with 10, 20 and 30 µM baicalein, respectively. The inhibition rate of MMP-9 was approximately 41.34%, 88.07% and 99.42% after 24 h of treatment with 10, 20 and 30 µM baicalein, respectively. The inhibition rate of u-PA was approximately 52.36%, 69.18% and 91.20% after 24 h of treatment with 10, 20 and 30 µM baicalein, respectively.

### Baicalein suppresses the expression and activity of MMP-2, MMP-9 and u-PA

Because the expression and activity of MMPs and u-PA are crucial to cell invasion, the expression and activity of MMP-2, MMP-9 and u-PA in MHCC97H cells that were exposed to different concentrations of baicalein were examined. Cells were treated with 0, 10, 20 and 30 µM baicalein for 24 h and then subjected to western blotting. Figure 4B and 4C show that baicalein significantly reduced the protein levels of MMP-2, MMP-9 and u-PA in a concentration-dependent manner compared with the control group.

Gelatin zymography was performed to assess the activity of MMP-2, MMP-9 and u-PA in cells treated with various concentrations of baicalein. As shown by gelatinolytic activity data, baicalein inhibited the activity of MMP-2, MMP-9 and u-PA in a concentration-dependent manner (Figure 4D). Quantification analysis indicated that MMP-9 activity was reduced by 41.2%, 76.02% and 91.81%, MMP-2 activity by 50.16%, 72.64% and 97.56% and u-PA activity by 62.73%, 85.02% and 96.34% in cells that were treated with 10, 20, and 30 µM of baicalein, respectively (Figure 4E).

### Baicalein promotes the expression of TIMP-1 and TIMP-2 in MHCC97H cells

Imbalances between MMPs and TIMPs play important roles in HCC progression and metastasis [21]; thus, the protein level of TIMP-1 and TIMP-2 in MHCC97H cells was assessed. MHCC97H cells were treated with 0, 10, 20 and 30 µM baicalein for 24 h and then subjected to western blotting. Figure 4F and 4G showed that baicalein significantly up-regulated the protein levels of TIMP-1 and TIMP-2 in a concentration-dependent manner.

### The ERK pathway is involved in the anti-metastatic mechanism of baicalein

**Figure 5:**
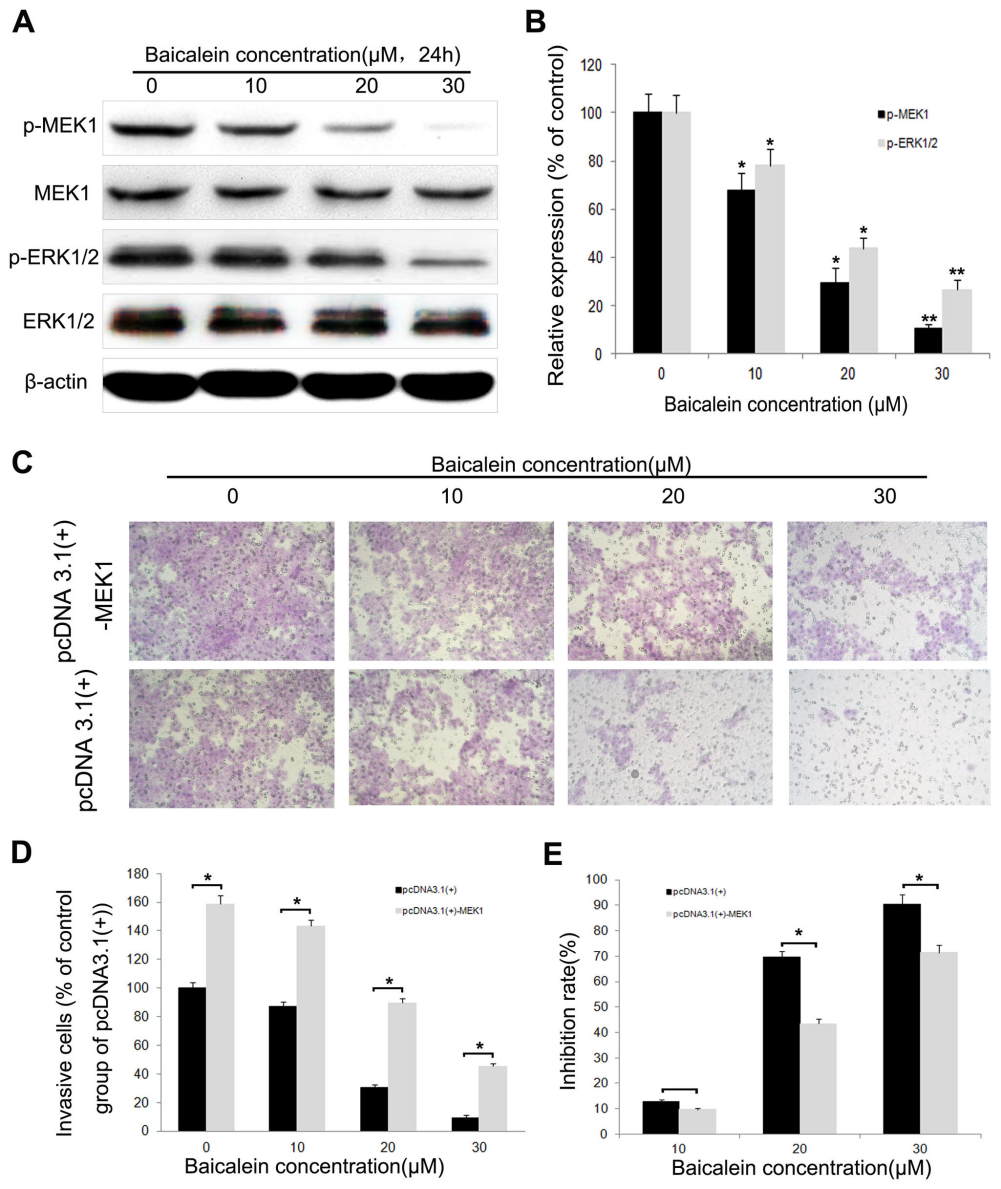
Effect of baicalein on the ERK pathway. (A) The protein levels of MEK1, p-MEK1, ERK1/2 and p-ERK1/2. (B) Phosphorylation densities of MEK1 and ERK1/2 were digitally scanned. (C) Transwell assays were performed to evaluate the anti-metastatic effects of baicalein on two groups of cells transfected with the indicated plasmids (transfected with the empty vector pcDNA3.1 (+) or with pcDNA3.1 (+)-MEK1, ‘pcDNA3.1(+)’ means the group transfected with an empty vector pcDNA3.1(+), ‘pcDNA3.1(+)-MEK1’ means the group transfected with a pcDNA3.1(+)-MEK1). (D) The percent invasion rate was expressed as a percentage of the control (the control group of cells transfected with an empty vector pcDNA3.1 (+)). (E) The inhibition rates of baicalein on two groups of cells. Values represent the means ± SD of three independent experiments performed in triplicate. *p < 0.05 and **p < 0.01 compared with the control group.

In human HCC cells, activation of ERK is required for the invasion process [22], and the mechanism is correlated with proteinases and their inhibitors [23–25]; thus, we investigated the effect of baicalein on the ERK pathway in MHCC97H cells. Western blotting showed that baicalein could reduce the phosphorylation of ERK1/2 and MEK1 in a concentration-dependent manner (Figure 5A and 5B).

To further verify the specific effect of baicalein on ERK pathway, we transfected MHCC97H cells with a plasmid (pcDNA3.1(+)-MEK1) expressing human MEK1 (Figure S1) and found that MEK1 reversed the inhibitory effect of baicalein on cell invasion (Figure 5C). The inhibition rate was approximately 13.2%, 70.1% and 91.3% after 24 h of treatment with 10, 20 and 30 µM baicalein, respectively, in the pcDNA3.1 (+) group (Figure 5E). In the pcDNA3.1 (+)-MEK1 group, the inhibition rate was 9.5%, 47.2% and 73.2%, respectively. We also found that after transfecting with an ectopically expressed form of MEK1, the invasive ability of MHCC97H cells was enhanced (Figure 5D).

**Figure 6:**
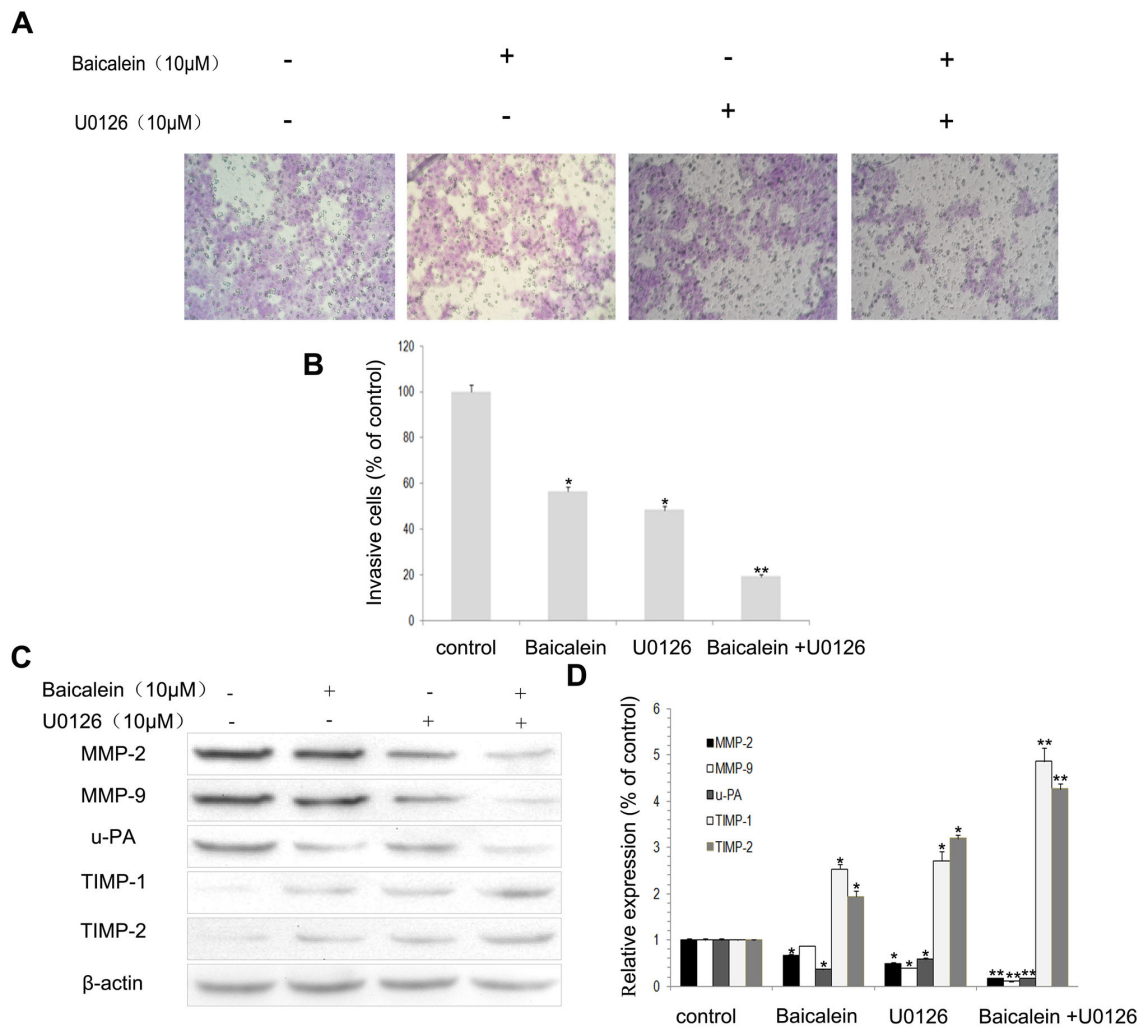
Effects of the ERK inhibitor (U0126) and baicalein on cell invasion and MMP-2, MMP-9, u-PA, TIMP-1 and TIMP-2 expression in MHCC97H cells. (A) Cells were pretreated with U0126 (10 μM) for 30 min and then incubated in the presence or absence of baicalein (10 μM) for 24 h. Cellular invasiveness was measured using the Boyden chamber invasion assay. (B) The percent invasion rate was expressed as a percentage of control. (C, D) MHCC97H cells were treated and then subjected to western blotting to analyze the protein levels of MMP-2, MMP-9, u-PA, TIMP-1 and TIMP-2. Values represent the means ± SD of three independent experiments performed in triplicate. *p < 0.05 and **p < 0.01 compared with the control group.

To further investigate whether the inhibitory effect of baicalein on cell invasion and MMP-2, MMP-9 and u-PA expression was correlated with inhibition of the ERK pathway, MHCC97H cells were pretreated with a ERK inhibitor (U0126; 10 μM) for 30 min and then incubated in the presence or absence of baicalein (10 μM) for 24 h. The cells with the indicated pre-treatment were then subjected to the *in vitro* invasion assay. The results show that treatment with U0126 and baicalein significantly reduced both cell invasion (Figure 6A and 6B) and also MMP-2, MMP-9 and u-PA protein expression (Figure 6C and 6D). However, the expression of TIMP-1 and TIMP-2 increased (Figure 6C and 6D). These results reveal that the inhibition of both cell invasion and MMP-2, MMP-9 and u-PA expression by baicalein occurs through the suppression of ERK pathways.

### Baicalein inhibits HCC metastasis in vivo

**Figure 7:**
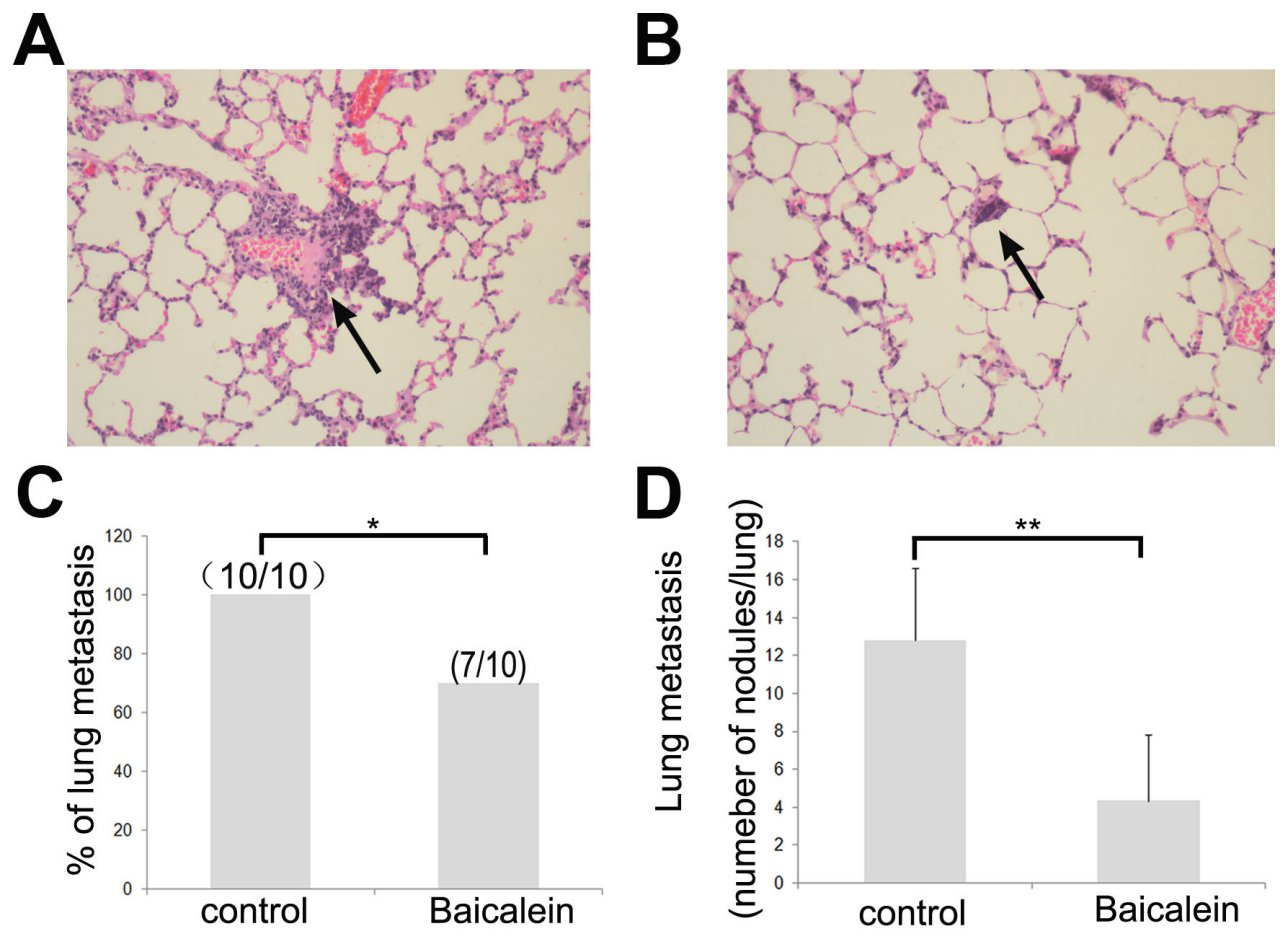
The anti-metastastic effects of baicalein on HCC *in vivo*. Microscopic findings of lung metastasis in the control group (A) and the treated group (B); black arrows show lung metastasis (100x). (C) The lung metastatic rate of the control and treated group. (D) The average number of lung metastasis in the control and treated group. *p < 0.05 and **p < 0.01 compared with the control group.

Overgrowth and metastasis are two major characteristics of malignant tumors [26]. Pulmonary metastasis occurs in 90% of HCC patients who have metastasis [27]. Here, we used an orthotopic transplantation nude mouse model of human HCC metastasis LCI-D20 [19] (100% tumorigenesis and pulmonary metastasis) to study the role and the mechanism of baicalein-mediated anti-metastatic effects on HCC *in vivo*. We treated orthotopic transplanted nude mice with diluent vehicle or baicalein. Compared to that of the control group, the size of the lung metastases of animals treated with baicalein were reduced (Figure 7A and B). The lung metastasis rate in control group was much higher than in the nude group treated with baicalein. In the control group, all of the mice had histologically proven lung metastasis (100%). In the treated group, 7 mice had histologically proven lung metastasis (70%; confidence interval [CI], 35 to 93%) (Figure 7C). The average number of lung metastases in the treated and control groups were 12.78 and 4.33, respectively. Lung metastases in the treated group decreased by 61.1% compared with those in the control group (Figure 7D).

## Discussion

The anti-tumor effect of baicalein has been confirmed in many cancers [28–30]. Recent studies showed that baicalein exerted anti-proliferative activity against HCC and lung cancer cells [31,32]. However, the anti-metastatic effect and the related mechanism(s) in HCC cells is not clear. This study revealed that baicalein significantly suppresses the invasive and metastatic ability of MHCC97H cells by regulation of the MMP/TIMP ratio via inhibition of the ERK pathway. According to our literature search, this is the first scientific report of the anti-metastatic effect of baicalein on HCC *in vivo*.

To elucidate the effect of baicalein on cell motility, cell migration and invasiveness were determined by a wound healing migration assay and the Boyden chamber invasion assay, respectively. The wound healing migration assay has been used very effectively for studying cellular migration across a 2D substratum. In the Boyden chamber invasion assay, cell invasion requires adhesion, proteolysis of ECM components and transmigration to penetrate the ECM. In these steps, expression of proteolytic enzymes, such as MMPs and u-PA, are crucial for ECM degradation [33,34]. In the present study, we demonstrated that baicalein significantly inhibits cell migration and invasion. Our study also showed that in MHCC97H cells that were treated with baicalein at non-toxic doses (no more than 50μM), cell migration and invasion were inhibited. These results implied that the inhibition of invasion by baicalein in MHCC97H cells was not due to cytotoxicity. To clarify the mechanism of action of baicalein, we investigated whether the inhibitory effect of baicalein on cell invasion is through regulation of the expression of MMPs and u-PA.

Metastasis is one of the leading causes of cancer-related death among HCC patients. Degradation of the ECM of blood or lymph vessels is critical to metastasis because loss of the ECM allows cancer cells to invade the blood or lymphatic system and spread to other tissues and organs. MMPs, especially MMP-2 and MMP-9, are responsible for breaking down the ECM [30,35]. In the plasminogen activation system, u-PA activity may be the most sensitive factor reflecting HCC invasion; thus, u-PA could be used as a strong predictor for the recurrence of HCC [36]. Therefore, MMP-2, MMP-9 and u-PA, which are secreted by invasive cancer cells, are considered to be important in cancer cell invasion and metastasis because of their roles in the degradation of the ECM [37–39]. In MMP-9-deficient mice, the number of metastatic colonies of B16-BL6 melanoma cells or Lewis lung carcinoma cells fell significantly when compared with normal mice [40]. By using chicken chorionallantoic membrane (CAM), a study showed that the cooperation of u-PA and MMP-9 is essential for tumor metastasis; however, the absence of MMP-9 or u-PA led to impaired invasive abilities in the tumor cells [41].

MMP activities can be inhibited by TIMPs to prevent extensive ECM degradation. The levels of TIMPs in serum and tissue were found to be significantly lower in HCC patients without metastasis than in those with metastasis [42]. In a mouse model of HCC, TIMP-1 plays an important part in tumor genesis and progression. TIMP-1 overexpression has been shown to suppress oncogene-induced hepatocarcinogenesis [43]. Our previous study showed that chrysanthemum indicum ethanolic extract (CIE) substantially suppressed the proliferation and invasiveness of a HCC cell line (MHCC97H), with a notable decrease in MMP-2 and MMP-9 expression and a simultaneous increase in TIMP-1 and TIMP-2 expression [30]. In the present study, we found that baicalein suppressed the expression and activity of MMP-2, MMP-9 and u-PA and simultaneously promoted TIMP-1 and TIMP-2 expression; thus, the MMP/TIMP balance was restored. These results indicate that the anti-metastatic effect of baicalein on MHCC97H cells was correlated with modulation of MMPs and their inhibitors (TIMPs).

The synthesis of proteinases and their inhibitors are regulated by multiple signaling cascades, including the ERK pathway [30,44–46]. The ERK pathway induces the expression of MMPs and thereby promotes the degradation of ECM proteins, which leads to tumor invasion [46]. Studies have shown that after ERK phosphorylation inhibition, the expression of MMP-2, MMP-9 and u-PA in HCC cells was down-regulated [11,47–49]. Activated MEK phosphorylates ERK1/2 on both a tyrosine and a threonine residue [50]. The only substrates for MEK that have been identified are ERK1 and ERK2 [51]. This tight selectivity in addition to the unique ability to phosphorylate both tyrosine and threonine residues is consistent with this kinase playing a central role in the regulation of ERK activation. To further explore the possible mechanism(s) of baicalein in the inhibition of HCC invasion, we determined the levels of phosphorylation of ERK1/2 and MEK1 in MHCC97H cells. The results demonstrated that the phosphorylation of ERK1/2 and MEK1 in cells treated with baicalein was significantly reduced relative to that in control cells.

In addition, we showed that introduction of ectopically expressed MEK1 reversed the inhibitory effect that baicalein has on the invasion capabilities of MHCC97H cells. The results also showed that treatment with an ERK inhibitor significantly reduced cell invasion and was accompanied by decreased MMP-2, MMP-9 and u-PA protein expression and increased TIMP-1 and TIMP-2 protein expression. These results suggest that baicalein inhibits the expression of MMP-2, MMP-9 and u-PA while elevating the expression of TIMP-1 and TIMP-2 via down-regulation of the ERK pathway. These results indicate that by down-regulating the ERK pathway, baicalein restores the MMPs/TIMPs balance in MHCC97H cells.

Finally, in the present study, the lung metastasis rate was found to be significantly decreased in the baicalein-treated nude mouse model LCID20. It has been suggested that baicalein may have a potential inhibitory effect on the cell invasion and metastastic capabilities of MHCC97H cells in vivo. In conclusion, this study demonstrated the inhibitory effect of baicalein on the invasion and metastastic capabilities of MHCC97H cells. Furthermore, the decrease in the expression of MMP-2, MMP-9 and u-PA induced by baicalein is attributed to an inhibition of the ERK signaling pathway. This mechanism may contribute to the inhibition of invasion and metastasis in MHCC97H cells by baicalein. These findings reveal a new potential therapeutic application of baicalein in anti-metastatic therapy for HCC.

## Supporting Information

Figure S1 **The relationship between the overexpression of MEK1 and ERK activity in MHCC97H cells transfected with pcDNA3.1** (**±)-MEK1.**
Western bloting analysis was performed to detect the expression of MEK1, p-MEK and ERK activity in three groups of MHCC97H cells: no transfection (control group ‘C’), cells transfected with an empty vector pcDNA3.1(±) (negative control group ‘N’), and cells transfected with a pcDNA3.1(±)-MEK1 (positive group ‘M’). (B) Quantification of the protein levels of MEK1, p-MEK1, ERK1/2 and p-ERK1/2. After transfecting with pcDNA3.1(±)-MEK1, the activity of ERK increased. Values represent the means ± SD of three independent experiments performed in triplicate. *p < 0.05 and **p < 0.01 compared with the control or negative group.
(TIF)
