# Supplementary figures and images for: Baicalein Inhibits the Invasion and Metastatic Capabilities of Hepatocellular Carcinoma Cells via Down-Regulation of the ERK Pathway
Source: PLoS One. 2013 Sep 6;8(9):e72927. doi: 10.1371/journal.pone.0072927 (PMC3765161; doi:10.1371/journal.pone.0072927)

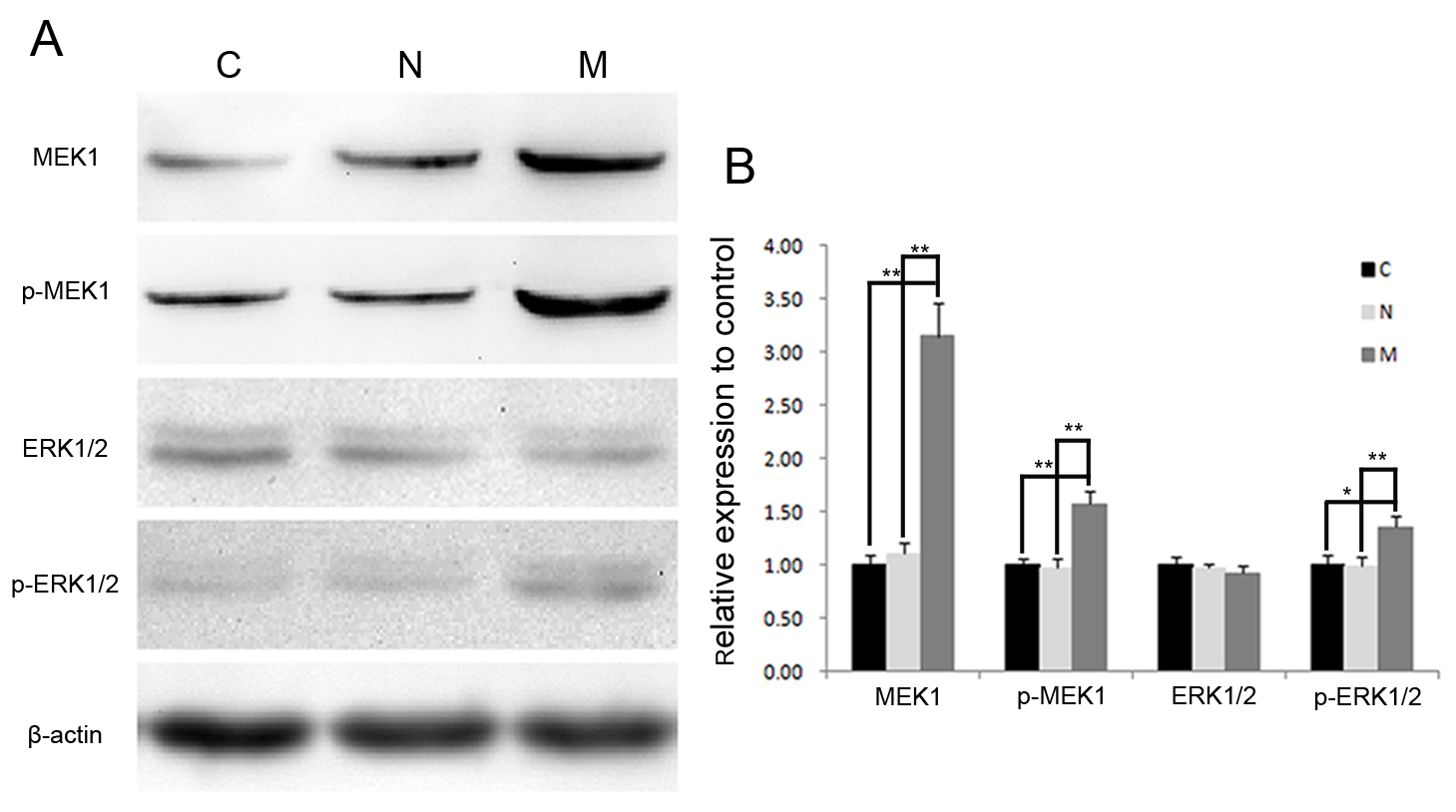

Supplement: Figure S1 — The relationship between the overexpression of MEK1 and ERK activity in MHCC97H cells transfected with pcDNA3.1 (±)-MEK1. Western bloting analysis was performed to detect the expression of MEK1, p-MEK and ERK activity in three groups of MHCC97H cells: no transfection (control group ‘C’), cells transfected with an empty vector pcDNA3.1(±) (negative control group ‘N’), and cells transfected with a pcDNA3.1(±)-MEK1 (positive group ‘M’). (B) Quantification of the protein levels of MEK1, p-MEK1, ERK1/2 and p-ERK1/2. After transfecting with pcDNA3.1(±)-MEK1, the activity of ERK increased. Values represent the means ± SD of three independent experiments performed in triplicate. *p < 0.05 and **p < 0.01 compared with the control or negative group. (TIF) [file pone.0072927.s001.tif]
